# Levels of Urinary Trypsin Inhibitor and Structure of Its Chondroitin Sulphate Moiety in Type 1 and Type 2 Diabetes

**DOI:** 10.1155/2018/9378515

**Published:** 2018-02-06

**Authors:** Antonio Junior Lepedda, Gabriele Nieddu, Silvia Rocchiccioli, Nadia Ucciferri, Michela Idini, Pierina De Muro, Marilena Formato

**Affiliations:** ^1^Dipartimento di Scienze Biomediche, University of Sassari, Sassari, Italy; ^2^Istituto di Fisiologia Clinica, National Research Council, Pisa, Italy

## Abstract

**Background:**

Diabetes mellitus is a global health problem representing the fifth leading cause of mortality and a major risk factor for cardiovascular diseases. In the last years, we reported an association among urinary trypsin inhibitor (UTI), a small proteoglycan that plays pleiotropic roles in many inflammatory processes, and both type 1 and 2 diabetes and developed a method for its direct quantitation and structural characterization.

**Methods:**

Urine from 39 patients affected by type 1 diabetes, 32 patients with type 2 diabetes, and 52 controls were analysed. UTI was separated from the main glycosaminoglycans physiologically present in urine by anion exchange chromatography, treated for chondroitin sulphate (CS) chain complete depolymerisation, and analysed for both UTI content and CS structure. UTI identification was performed by nano-LC-MS/MS analysis.

**Results:**

We evidenced increased UTI levels, as well as reduced sulphation of its CS moiety in association with diabetes, regardless of both age and medium-term glycaemic control. Furthermore, no association between UTI and albumin excretion rate was found.

**Conclusions:**

Evidences suggest that UTI levels are not directly correlated with renal function or, otherwise, that they may increase before the onset of renal impairment in diabetes, representing a potential marker for the underlying inflammatory condition.

## 1. Introduction

**Figure 1:**
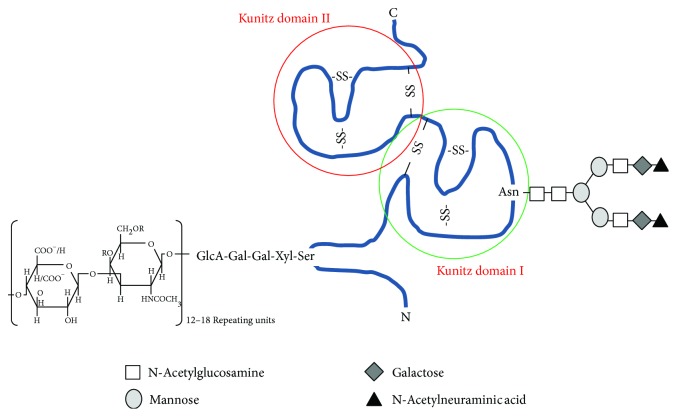
Schematic drawing of urinary trypsin inhibitor structure. R=H/SO_3_^−^.

Urinary trypsin inhibitor (UTI) is a small proteoglycan (PG), with inhibitory activity against serine proteases, resulting from excretion of plasma bikunin into urine [1]. Despite that UTI was purified in 1955 for the first time [2] and several studies on its structure and levels have been performed since the 1950s, its biological function has not been fully understood yet [3]. It is composed of a protein moiety, a low-sulphated chondroitin sulphate (CS) chain, O-linked to the serine 10, as well as an oligosaccharide, N-linked to the asparagine 45 of the protein moiety (Figure 1). The polypeptide portion consists of 147 amino acid residues folded in two Kunitz-type domains (7 kDa each) containing three disulphide bonds, a connecting peptide as well as N- and C-terminal moieties of 10–25 amino acid residues each [4]. The molecular mass of the whole proteoglycan is about 25-26 kDa, being the protein core, the CS moiety, and the oligosaccharide chains 16 kDa, 7 kDa, and 2 kDa, respectively, as reported by various studies and confirmed by ultracentrifugation methods [1, 3–5]. However, because of CS chain extended conformation, bikunin behaves like a globular protein of about 67 kDa by gel filtration and has an apparent molecular mass of 35–45 kDa by SDS-PAGE [6]. CS chain is composed of 12–18 disaccharide repeating units, consisting of glucuronic acid and N-acetyl galactosamine (GalNAc), which may be sulphated either in position C4 or in C6 [7, 8]. Four monosaccharides (Xyl-Gal-Gal-GlcA) connect this glycosaminoglycan (GAG) to bikunin [8].

About 90–98% of bikunin occurs in plasma as a subunit of interalpha inhibitor family molecules, linked via an ester bond between C6 of a nonsulphated GalNAc residue of the CS chain and the *α*-carbon of the C-terminal amino acid residue of one or two polypeptides [9], called the heavy chains, to form interalpha inhibitor and prealpha inhibitor, respectively [1].

Although its main activity is the inhibition of various serine proteases such as trypsin, chymotrypsin, elastase, granzyme K, cathepsin G, acrosin, and plasmin [3, 4, 10], many studies provided evidences also on its role in many regulatory mechanisms, such as inhibition of IL-8 gene expression induced by lipopolysaccharide [10], neutrophil release of elastase [11], mast cell release of histamine [12], urolithiasis [13], stabilization of the lysosomal membrane [14], stimulation of fibroblast growth [15], and regulation of smooth muscle contraction by modulating calcium intracellular levels [16], as well as in supporting the formation of the hyaluronan-containing extracellular matrix [17] and in inhibiting the formation of kidney stone [18].

Furthermore, bikunin was proven to have a protective role in several inflammatory processes, by preventing protease-activated receptor activation on cell surfaces [19], by inhibiting blood coagulation through its action on plasmin and blood coagulation factors [20], and by protecting acinar and endocrine pancreatic cells from self-digestion [21], thus providing protection against pathologies such as diabetes, kidney diseases, cancer, bacterial and viral infections, and vascular diseases [21].

Several studies reported that UTI/bikunin levels can increase up to 10-fold following both acute and chronic inflammatory diseases [22], bladder carcinoma [23], brain contusion [24], disseminated cancers [25], acute hepatitis [26], Fabry's disease [27], Crohn's disease, arthritis, pericarditis, deep vein thrombosis, fibromyalgia, asthmatiform bronchitis [28, neoplasia, and kidney diseases [4]. Besides, we reported variations of UTI/GAGs levels in pathological conditions such as chronic glomerulonephritis [29, 30], type 1 and 2 diabetes [31–35], systemic lupus erythematosus [36] and following kidney transplantation [37]. In human plasma, the concentration of bikunin is 4–7 *μ*M, of which only 2–10% is in free form, while in urine UTI levels are about 0.03–0.05 *μ*M [4].

To date, UTI quantitation in urine is performed mainly by means of enzyme inhibition assays or immunological detection [38]. Both approaches may be affected by low specificity and/or sensibility. Recently, we developed a method for a direct quantitation and structural characterization of UTI protein moiety, starting from very low quantities of specimen. To achieve this goal, we merged classical chromatographic methods, applied for GAG/PG purification [27, 30, 31, 33, 34, 36], with image analysis of SDS-PAGE profiles, which had been proven useful in quantifying protein microquantities from different sources [39]. Furthermore, nano-LC-MS/MS analysis on tryptic-digested UTI bands was performed for protein identification and characterization.

The aim of the present study was to characterize the CS moiety of UTI in diabetic patients by fluorophore-assisted carbohydrate electrophoresis (FACE) analysis, applying a preanalytical step for UTI purification. Furthermore, the obtained results allowed us to validate previous preliminary data on both type 1 and 2 diabetes mellitus (T1DM and T2DM) suggesting UTI as a potential useful marker of these two chronic inflammatory conditions [32].

## 2. Methods

### 2.1. Sample Collection

Twenty-four-hour urine samples were collected from 39 patients affected by type 1 diabetes (age 31.79 ± 10.90), 32 patients with type 2 diabetes (age 64.50 ± 7.50), and 52 controls (age 36.25 ± 19.34) and immediately centrifuged at 5000 ×g for 15 minutes before storing at −20°C until analysis. Each sample was assessed for both urinary creatinine, carried out by the Jaffè method (Sentinel Diagnostics, Italy), and twenty-four-hour albumin excretion rate (AER), assessed by an immunoturbidimetric method (Roche Diagnostics, Germany). All diabetic patients were normoalbuminuric with an AER lower than 30 mg/24 hours except for three microalbuminuric patients. Fasting glycaemia was outside the target range (70–130 mg/dL) being 163.44 ± 29.99 mg/dL and 145.10 ± 22.78 mg/dL for T1DM and T2DM, respectively, whereas glycated haemoglobin (HbA1c%) was 7.51 ± 0.95 and 6.70 ± 0.86, indicating a poor long-term control of blood glucose in both groups.

Informed consent was obtained before enrolment. Institutional Review Board approval was obtained. The study was conducted in accordance with the ethical principles of the current Declaration of Helsinki.

### 2.2. UTI Purification and Analysis

**Figure 2:**
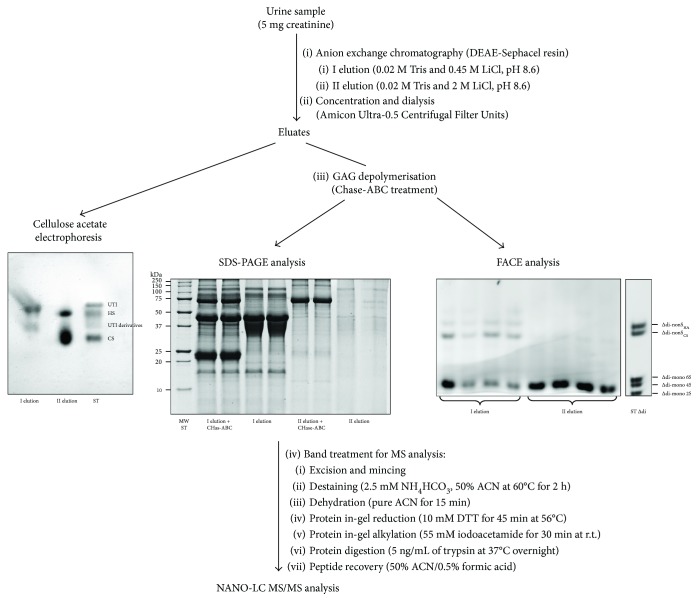
Flow chart showing the adopted methods for UTI purification (anion exchange chromatography) and analysis of both protein core (SDS-PAGE followed by MS analysis) and chondroitin sulphate moiety (fluorophore-assisted carbohydrate electrophoresis analysis). Cellulose acetate electrophoresis was performed to assess effectiveness of UTI purification. I elution: UTI and UTI derivatives; II elution: heparan sulphate (HS) and free highly charged chondroitin sulphate (CS).

UTI was purified according to a method recently developed by our research group [32] with slight modifications (Figure 2). Briefly, two elution fractions were obtained by anion exchange chromatography; the first one contained UTI and UTI derivatives, while the latter contained free highly charged GAGs, that is, HS and CS, as assessed by electrophoresis on cellulose acetate plates. Following chondroitin ABC lyase (Chase-ABC) depolymerisation of CS moiety, UTI fraction was analysed for both UTI content, by SDS-PAGE, and CS detailed structure, by FACE. UTI band obtained by SDS-PAGE was identified by MS analysis.

### 2.3. Anion Exchange Chromatography

A volume of urine corresponding to 5 mg of creatinine (usually ranging from 2 to 8 mL) was loaded into a chromatography column (Econo-Column Chromatography Columns, 0.5 × 20 cm, Bio-Rad Laboratories, California, USA) packed with DEAE-Sephacel anion exchange resin (GE Healthcare Life Sciences, United Kingdom) and equilibrated with 0.02 M Tris buffer, pH 8.6, containing 0.15 M NaCl, which was also used to wash away from the column all the by-products and impurities contained in urine samples until absorbance at 280 nm was lower than 0.05. Two consecutive gravity feed elution steps were performed using 0.02 M Tris buffer, pH 8.6, containing 0.45 M LiCl, and 0.02 M Tris buffer, pH 8.6, containing 2 M LiCl, to elute separately UTI-containing fraction (first elution) from highly charged urinary GAGs (second elution). Both fractions were immediately concentrated and dialysed by means of Amicon Ultra-0.5 Centrifugal Filter Units (Millipore, MA, USA), according to the manufacturer's instructions, for further analyses.

### 2.4. Cellulose Acetate Electrophoresis Analysis

To evaluate GAG composition, aliquots of both eluted fractions were resolved by discontinuous electrophoresis on Titan III-H cellulose acetate plates (6.0 × 7.5 cm, Helena BioSciences, United Kingdom), according to Cappelletti et al. [40]. This technique, combined with the differential susceptibility of GAGs to precipitation by organic solvents, allows for optimal and rapid separation of intact GAGs with high resolution and sensitivity (10 ng detection limit). GAG separation was carried out in 0.25 M barium acetate buffer, pH 5.0, by three electrophoretic steps as previously described [27]. Electrophoretic profiles were detected following 0.1% (*w*/*v*) alcian blue staining. Images were acquired by means of a GS-800 calibrated densitometer (Bio-Rad Laboratories, California, USA) and analysed by using Quantity One v4.6.3 software (Bio-Rad Laboratories, California, USA).

### 2.5. Chondroitin Sulphate Depolymerisation

Following concentration and dialysis, UTI fraction was diluted with 0.5 M ammonium acetate 5X buffer, pH 8.0, and incubated overnight at 37°C with 0.025 U of Chase-ABC (Sigma-Aldrich, MO, USA), allowing for chondroitin sulphate complete depolymerisation into constituent disaccharide units with an unsaturation between C4 and C5 of hexuronic acid (∆-disaccharides). Subsequently, samples were split in two aliquots and analysed for both UTI content, by SDS-PAGE, and CS detailed structure, by FACE.

### 2.6. SDS-PAGE Analysis

Samples were diluted with 4X Laemmli buffer, consisting of 250 mM Tris, pH 6.8, 8% SDS (*w*/*v*), 8% dithiothreitol (DTT) (*w*/*v*), 40% glycerol (*v*/*v*), and 0.0008% bromophenol blue (*w*/*v*), boiled for 5 minutes, and resolved by Tris-glycine SDS-PAGE, through 1 mm thick 15% T and 3% C running gel. Electrophoresis was carried out at 50 V for 15 minutes and then at 150 V, until the bromophenol dye front reached the lower limit of the gel, in a MiniProtean II cell vertical slab gel electrophoresis apparatus (Bio-Rad Laboratories, California, USA). Following Coomassie brilliant blue G-250 staining, gels were acquired at 63 *μ*m resolution using a GS-800 calibrated densitometer (Bio-Rad Laboratories, California, USA) and analysed by means of Quantity One software, v 4.6.3 (Bio-Rad Laboratories, California, USA) [39]. For UTI quantitation, a calibration curve was set (*y* = 1.337*x* − 0.1868, *R* ^2^ = 0.9943) by loading known quantities of highly purified CS-free UTI, ranging from 0.25 to 4 *μ*g. The linear response between band intensity, expressed as optical density, and UTI levels allowed for accurate quantitation in the considered range. UTI concentration in each sample was normalized for creatinine content.

### 2.7. UTI Identification by Nano-LC-MS/MS Analysis

Nano-LC-MS/MS analysis on tryptic-digested UTI bands was performed for protein identification and characterization as previously described [31, 32]. Briefly, spots were excised, destained, and dehydrated. Rehydrated spots were in gel reduced, alkylated, and trypsin digested. Recovered peptides were purified through a C18 column, and 2 *μ*L was injected into a nano-HPLC system (Eksigent, ABSciex, USA). Peptides were separated through a C18 PepMap-100 column (3 *μ*m, 75 *μ*m × 250 mm, Thermo Scientific, USA) in a 70-minute linear gradient from 5% of 0.1% formic acid to 40% of acetonitrile/0.1% formic acid. HPLC was directly coupled to a TripleTOF™ 5600 mass spectrometer (ABSciex, USA), and MS/MS data were processed with ProteinPilot™ Software (ABSciex, USA).

### 2.8. FACE Analysis

Structural analysis on CS moiety was performed on samples from 10 T1DM and 10 T2DM patients and 10 healthy controls, as previously described [41]. ∆-Disaccharide units obtained by depolymerisation of CS chain from UTI fraction were fluorotagged by reductive amination with 2-aminoacridone (AMAC), in the presence of sodium cyanoborohydride (NaBH_3_CN), as previously described by Calabro et al. [42]. Briefly, 40 *μ*L of 12.5 mM AMAC solution in glacial acetic acid/DMSO (3 : 17 *v*/*v*) was added to Chase-ABC-treated lyophilized sample and incubated for 15 minutes, at room temperature, followed by 40 *μ*L of 1.25 M NaBH_3_CN and overnight incubation at 37°C. After derivatization, 20 *μ*L of glycerol was added to each sample prior to electrophoresis, which was performed in a MiniProtean II cell vertical slab gel electrophoresis apparatus (Bio-Rad Laboratories, California, USA) as previously described by Karousou et al. [43]. 5 *μ*L of each sample was resolved through 25% T and 7.5% C polyacrylamide running gels prepared in 187.5 mM Tris-borate and 187.5 mM Tris-HCl buffer, pH 8.8. 5% T and 15% C stacking gels were prepared in 0.36 M Tris-HCl buffer, pH 8.8. 5 *μ*L of bromophenol blue dye was run in the first lane to monitor the electrophoresis front. The run was carried out in 0.15 M Tris-borate buffer, pH 8.8, at 400 V and 4°C until the dye reached the bottom of the gel. Gels were then acquired by UV transillumination using the Gel Doc XR System (Bio-Rad) and analysed with Quantity One 4.6.3 (Bio-Rad Laboratories, California, USA). Band identification was achieved by comparing their electrophoretic mobility with standard ∆-disaccharides, run in the same gel. A calibration curve was set up (*y* = 909.14*x* − 9891.9, *R* ^2^ = 0.9996), allowing for accurate ∆-disaccharide quantitation in each sample, by loading known quantities (from 25 to 800 ng) of AMAC-derivatized ∆-disaccharides obtained by depolymerisation of a highly purified UTI sample assayed for uronic acid (UA) content, according to the carbazole method by Bitter and Muir [44]. CS concentration in each sample was normalized for creatinine content.

### 2.9. Statistical Analysis

Statistical analyses were performed using Sigma Stat 3 software package (Systat Software). UTI concentration values were reported as median and interquartile range, as normality test failed. Mann–Whitney rank sum test was performed to evaluate differences among the three groups, while correlations between UTI levels and age, UTI levels and glycated haemoglobin, and UTI levels and microalbuminuria were assessed by Spearman's correlation. Significance was set at *p* < 0.05.

## 3. Results

Both purification and quantitation of UTI were performed according to a method recently published [31, 32], with some modifications (Figure 2). In particular, as the purpose of this work was to analyse at a structural level UTI-CS moiety, we performed two elution steps that allowed the separation of UTI fraction (first elution) from the highly charged GAG fraction (second elution), containing HS and a normosulphated CS with different origin from UTI-CS. Then, both fractions were concentrated, dialysed, and treated with Chase-ABC for CS complete depolymerisation into ∆-disaccharide units.

A half of each sample was subjected to SDS-PAGE analysis for UTI quantitation and identification by nano-LC-MS/MS analysis (Table 1, see Supplementary Material 1 for complete MS information), while the other one, following AMAC derivatization, was analysed by means of FACE for CS content and structure. Since all data were normalized for creatinine content, the method was effective in analysing also randomly collected urine samples in a wide range of concentrations. Furthermore, an estimation of UTI-CS chain length was performed as the ratio between the moles of disaccharides and the moles of UTI, considering 15.974 kDa as UTI molecular weight calculated through ExPASy Compute pI/Mw tool (http://www.expasy.org), according to the sequence reported by Xu et al. [45].

**Figure 3:**
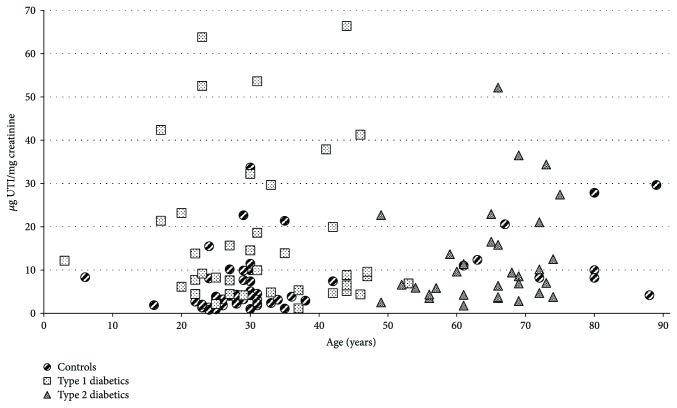
Scatter plot showing UTI levels in patients with type 1 diabetes (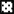) and type 2 diabetes (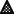) and healthy controls (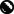) in relation to age.

Data obtained from patients affected by both type 1 and type 2 diabetes were compared with those from a healthy control group (Table 2). We evidenced higher levels of UTI, expressed as *μ*g protein/mg creatinine, in both T1DM and T2DM patients with respect to controls (*p* = 0.001 and *p* = 0.006, resp.), whereas no differences were found between the two groups of patients (*p* = 0.160) (Figure 3). With regard to the glycosaminoglycan moiety of UTI, CS levels, expressed as *μ*g UA/mg creatinine, were found significantly higher in both T1DM and T2DM patients with respect to controls (*p* = 0.005 and *p* = 0.041, resp.). Besides, a lower sulphation degree of CS chains, expressed as ratio between ∆di-mono 4S and total disaccharides (∆di-mono 4S + ∆di-non S), was observed in both classes of patients compared to controls (*p* = 0.046 and *p* = 0.021 for T1DM and T2DM, resp.), while no differences were found between T1DM and T2DM patients (*p* = 0.873). Finally, no differences in CS chain length were evidenced between both classes of patients and controls (*p* = 0.967, *p* = 0.096, and *p* = 0.473, for T1DM versus controls, T2DM versus controls, and T1DM versus T2DM, resp.) as further corroborated by the strict positive correlation between levels of UTI protein core and CS reported for T1DM (*p* < 0.001), T2DM (*p* < 0.001), and the totality of samples (including controls) (Table 3).

To evaluate any possible association among UTI and medium-term glycaemic control or renal function, we performed Spearman's correlation tests evidencing no correlation neither with glycated haemoglobin nor with albumin excretion rate (Table 4). Finally, no correlation was evidenced between UTI levels and age in controls, T1DM, and T2DM and in the totality of samples (Table 5).

## 4. Discussion

Diabetes mellitus is a huge global health problem representing the fifth leading cause of mortality and a major risk factor for cardiovascular diseases, such as coronary artery disease, stroke, and peripheral vascular disease [46]. T1DM results from the autoimmune destruction of the insulin-producing *β* cells of Langerhans islets; it is usually diagnosed in children and young adults and represents less than 10% of all cases of diabetes. T2DM, or adult-onset diabetes, represents over 90% of cases of diabetes mellitus and is characterized by hyperglycaemia caused by insulin resistance. A major issue of T2DM is that its diagnosis is frequently made years after disease onset, when vascular complications are already present in most patients [47]. Since patients with diabetes are at increased risk of microvascular and macrovascular complications [47], identification of early diagnostic markers to be associated with the current standard tests is mandatory to slow disease progression and reduce adverse outcomes.

The association between diabetes and GAG/PG excretion in urine has been extensively studied [48]. Except for a few studies that report no change or even a reduced excretion of GAGs/PGs, a plethora of papers evidence an increased excretion as a whole (in most of them only a quantitative analysis was performed) and in particular of HS in both type 1 and 2 diabetes, often in association with diabetic nephropathy [48]. In the last years, we reported an association among UTI and both type 1 and 2 diabetes [31–35] suggesting UTI as a promising marker for the chronic inflammation resulting from diabetic condition [31, 32]. Despite the numerous studies on both UTI structure and function, several aspects related to its structural modifications following inflammation and their relevance for a biological and diagnostic point of view remain to be elucidated. In this respect, Mizon et al. demonstrated that the CS chain of bikunin increases in size in inflammatory diseases [49]. Furthermore, Capon et al. evidenced that, in acute inflammation, the CS chain is both longer and undersulphated [50].

The aims of this study were both to characterize at a structural level the CS moiety of UTI, which has not been analysed so far in relation to diabetic condition, and to strengthen our previous preliminary results on both T1DM and T2DM, suggesting UTI as a potential useful marker of these two chronic inflammatory conditions.

Hence, we evidenced that diabetes may result in UTI levels increase, as well as in changes in CS chain sulphation degree. In this respect, we did not evidence any correlation among UTI levels, age and glycated haemoglobin ruling out these parameters from the potential confounders. Furthermore, no association between UTI and albumin excretion rate was found, suggesting that UTI levels are not directly correlated with renal function or, otherwise, that they may increase before the onset of renal impairment.

With regard to CS chain sulphation degree, UTI was undersulphated in both T1DM and T2DM patients. The sulphation balance and pattern of CS on specific carbon residues are tightly regulated during development, injury, and disease, with the temporal and spatial expression of different chondroitin sulfotransferase isoforms [51].

Glycosaminoglycan/proteoglycan biosynthesis and turnover require many enzymatic activities and are finely regulated by molecules such as hormones, cytokines, and growth factors [52, 53]. In particular, it is known that in diabetic patients, there is an increased renal production of TGF-*β* that is a potent modulator of extracellular matrix (ECM) proteoglycan synthesis and represents a potential link between hyperglycaemia and the accelerated development of atherosclerosis in diabetes [54].

Improving diagnosis and follow-up of both T1DM and T2DM is mandatory, as they may result in microvascular and macrovascular complications, as well as in a significant increase in cardiovascular risk [47]. In this study, both levels and structure of UTI have been found associated with T1DM and T2DM and may represent potential markers for the underlying inflammatory condition. As far as we know, the relationship between UTI-CS structure and diabetes has not been investigated yet. Therefore, we can only speculate that similar pathways involved in ECM proteoglycan remodelling could affect UTI biosynthesis in the liver. The applied method was proven valuable in both UTI purification from free highly charged CS and quantitative/structural analyses, also starting from small sample volumes or randomly collected samples. Therefore, it may represent a promising tool in monitoring the onset and the progression of type 1 and type 2 diabetes. The mechanisms underlying UTI modifications in diabetes as well as their pathophysiological role are not yet known and surely deserve further studies.

## Figures and Tables

**Table 1:** UTI identification by nano-LC-MS/MS analysis in the 3 groups analysed.

	Paragon score	% Cov (95)	Accession number	Entry name	Protein name	Species	Peptides (95%)
Controls	110.11	38.64	P02760	AMBP_HUMAN	Protein AMBP	HUMAN	149
T1DM	167.84	57.1	P02760	AMBP_HUMAN	Protein AMBP	HUMAN	337
T2DM	123.92	41.76	P02760	AMBP_HUMAN	Protein AMBP	HUMAN	218

Each spot was analysed separately and MS/MS data from the same group were treated as replicates for database analysis. % Cov = coverage percentage with at least 95% confidence. Peptides = identified peptides with at least 95% confidence. AMBP = alpha-1-microglobulin/bikunin precursor.

**Table 2:** UTI levels and CS moiety structure in T1DM, T2DM, and healthy controls.

	Controls	T1DM	T2DM	T1DM versus controls	T2DM versus controls	T1DM versus T2DM
UTI protein core levels (*μ*g prot./mg creatinine)^a^	4.093^d^(2.326–9.920)	9.612^d^(5.532–22.738)	7.792^d^(4.304–16.181)	**0.001** ^b^	**0.006** ^b^	0.160^b^
CS levels (*μ*g UA/mg creatinine)^a^	1.251^e^(1.041–1.413)	1.940^e^(1.459–5.949)	1.514^e^(1.315–2.286)	**0.005** ^b^	**0.041** ^b^	0.162^b^
Sulphation degree (∆di-mono 4S/∆di-mono 4S + ∆di-non S)^a^	53.113%^e^(47.360–55.338)	42.612%^e^(35.734–49.263)	44.712%^e^(36.633–50.073)	**0.046** ^c^	**0.021** ^c^	0.873^c^
Chain length (moles of ∆di/moles of UTI)^a^	10.951^e^(9.010–12.780)	8.967^e^(8.498–23.598)	12.764^e^(12.212–15.502)	0.967^b^	0.096^b^	0.473^b^

^a^Median and interquartile ranges (in parenthesis) are reported. ^b^ *p* values, obtained by the Mann–Whitney Rank Sum tests, are reported. ^c^ *p* values, obtained by *t*-tests, are reported. ^d^Data obtained from 52 controls, 39 type 1 diabetics, and 32 type 2 diabetics. ^e^Data obtained from 10 controls, 10 type 1 diabetics, and 10 type 2 diabetics. Significant differences are reported in bold (*p* < 0.05).

**Table 3:** Spearman's correlation tests between levels of UTI protein core and CS moiety.

UTI protein core versus CS	Corr. coefficient	*p* value
Controls	0.376	0.284
Type 1 diabetics	**0.897**	<0.001
Type 2 diabetics	**0.911**	<0.001
All (including controls)	**0.763**	<0.001

Positive correlation between values that tend to increase together is indicated in bold.

**Table 4:** Spearman's correlation tests between UTI protein core levels and glycated haemoglobin and between UTI protein core levels and AER.

UTI versus HbA1c%	Corr. coefficient	*p* value
T1DM	0.318	0.087
T2DM	−0.152	0.500
UTI versus AER		
T1DM	−0.156	0.457
T2DM	−0.004	0.986

**Table 5:** Spearman's correlation tests between UTI protein core levels and age.

UTI versus age	Corr. coefficient	*p* value
Controls (under 50 years old)	0.103	0.510
Controls (over 50 years old)	0.178	0.646
Type 1 diabetics	−0.070	0.673
Type 2 diabetics	0.243	0.181
All (including controls)	0.088	0.330

## Supplementary Materials

Supplementary Materials Supplementary Material 1: complete MS data about UTI identification by nano-LC-MS/MS analysis.
